# Supplementary material for: Effects of Orientation and Dispersion on Electrical Conductivity and Mechanical Properties of Carbon Nanotube/Polypropylene Composite
Source: Polymers (Basel). 2023 May 19;15(10):2370. doi: 10.3390/polym15102370 (PMC10220618; doi:10.3390/polym15102370)
Supplement: Supplementary file 1 [file polymers-15-02370-s001.zip › polymers-2406429-supplementary.pdf]

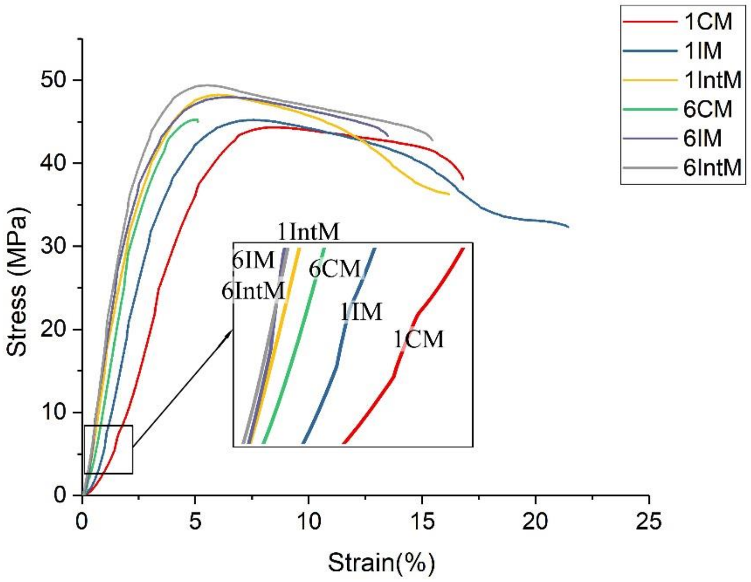

Figure S1. Stress-strain curves of PP/CNT composites

IntM samples formed by high shear stress can get a higher modulus at any CNT content. While the modulus of IM and CM can be increased by CNTs.
